# The spermatogenic process of the common vampire bat *Desmodus rotundus* under a histomorphometric view

**DOI:** 10.1371/journal.pone.0173856

**Published:** 2017-03-16

**Authors:** Danielle Barbosa Morais, Luciano Carlos Heringer Porcaro Puga, Tarcízio Antônio Rêgo de Paula, Mariella Bontempo Duca Freitas, Sérgio Luis Pinto da Matta

**Affiliations:** 1 Department of Morphology, Federal University of Rio Grande do Norte, Natal, Rio Grande do Norte, Brazil; 2 Department of Veterinary, Federal University of Viçosa, Viçosa, Minas Gerais, Brazil; 3 Department of Animal Biology, Federal University of Viçosa, Viçosa, Minas Gerais, Brazil; 4 Department of General Biology, Federal University of Viçosa, Viçosa, Minas Gerais, Brazil; University Hospital of Münster, GERMANY

## Abstract

Among all bat species, *Desmodus rotundus* stands out as one of the most intriguing due to its exclusively haematophagous feeding habits. However, little is known about their spermatogenic cycle. This study aimed at describing the spermatogenic process of *common vampire bats* through testicular histomorphometric characterization of adult specimens, spermatogenic production indexes, description of stages of the seminiferous epithelium cycle and estimative of the spermatogenic process duration. Morphometrical and immunohistochemical analyzes for bromodeoxiuridine were conducted under light microscopy and ultrastructural analyzes were performed under transmission electron microscopy. Vampire bats showed higher investment in gonadal tissue (gonadosomatic index of 0.54%) and in seminiferous tubules (tubulesomatic index of 0.49%) when compared to larger mammals. They also showed a high tubular length *per* gram of testis (34.70 m). Approximately half of the intertubular compartment was found to be comprised by Leydig cells (51.20%), and an average of 23.77x10^6^ of these cells was found *per* gram of testis. The germline cells showed 16.93% of mitotic index and 2.51% of meiotic index. The overall yield of spermatogenesis was 60% and the testicular spermatic reserve was 71.44x10^7^ spermatozoa *per* gram of testis. With a total spermatogenesis duration estimated at 37.02 days, vampire bats showed a daily sperm production of 86.80x10^6^ gametes *per* gram of testis. These findings demonstrate a high sperm production, which is commonly observed in species with promiscuous mating system.

## Introduction

Bats vary widely in form, ecological requirements and reproductive adaptations. On this last topic, male’s response to female’s reproductive cycle is expressed in a wide variety of reproductive patterns, although most male’s responses seem triggered by the female’s sexual readiness. Reproductive patterns are usually more flexible in species with constant nutritional supply, i.e., less pronounced seasonal events [1].

The common vampire bat *Desmodus rotundus* is endemic in Latin America, and is one of the three bat species which feeds exclusively on blood. Unlike the others, *Desmodus* is quite common and is specialized on bovine blood, being a potential vector of rabies virus [2–4]. This species reproduces continuously [1]. Their mating system shows a promiscuous pattern, where the groups are composed of 2 to 10 males and 8 to 12 females [1, 5]. Females are characterized as polyestrous [1, 6, 7]. Although abundant in the Neotropics, little information is available on their reproductive cycle regarding the reproduction of males. Although some histochemical and structural characterization of testicular components have been described [8, 9], no studies were found quantifying the testicular activity and spermatogenesis cycle in this species.

Given their ecological importance to the maintenance of biodiversity [2] and due to public health management and control, knowledge of the reproductive cycle of males might be helpful to achieve a rational management of the species. Recent studies on the spermatogenic process in other Chiropteran species have shown interesting characteristics of these animals when compared to other mammals, such as high levels of sperm production and short spermatogenical cycle [10–12], which has given a prominent role to bats regarding their reproductive capacity.

Thus, this study aimed describing the complete spermatogenic process of *D*. *rotundus*, through the analysis of the testicular histomorphometry, calculation of indicative indexes of sperm production, characterization of the cells’ ultrastructure at the seminiferous epithelium and the estimation of the duration of the spermatogenic process.

## Material and methods

### Study area and animal collections

The animals were collected in an abandoned mine of malacacheta exploration, in the city of Juiz de Fora, Paula Lima district, Minas Gerais state, Brazil (21°35'11,1"S and 43°21'49,0"W) with an approximate altitude of 720 m (source: INMET—National Institute of Meteorology, Brazil). Collections happened in June 2011, using mist nets (7x2 m) near the animals’ roost (license number 214887-1/SISBIO/IBAMA). After capture of the animals in their natural environment, during its return for the shelter after foraging, the animals were kept in a cage for birds protected from light. In the morning following the night of capture the animals were transported to the city of Viçosa, Minas Gerais (mean distance of 187 km).

Six adult males (*Desmodus rotundus*) were used in this study. Adult animals were identified based on the fusion of the epiphyseal cartilage of the fourth finger, at the metacarpal-phalangeal junction [13]. Bats were fed with bovine blood taken from healthy cows from Department of Veterinary of Federal University of Viçosa (UFV), and treated with anti-coagulant in the night of capture. The blood was obtained during routine veterinary care, without cows’ euthanasia. Since the animals’ collection to euthanasia, water was provided *ad libitum*. Euthanasia was held the following morning. The euthanasia was performed by an intra-peritoneal injection of sodium pentobarbital (40 mg/kg) (Nembutal, Sigma–Aldrich, St. Louis, MO, USA) followed by a saturated solution of potassium chloride. All experimental procedures were conducted in accordance with the recommendations of the National Council for Animal Experimentation Control (CONCEA), and were approved by the Ethics Committee on Animal Use (CEUA) of Federal University of Viçosa (document number 93/2011). All efforts were made to minimize animal suffering.

### Histological processing

The testes were fixed by immersion in Karnovsky solution [14] for 24 h and transferred to 70% ethanol. Both testes and the tunica albuginea of one of them were removed and weighed, being the tunica albuginea weight diminished from the gonad weight to calculate the volume of the testicular parenchyma.

Testicular fragments were dehydrated in increasing ethanol series for inclusion either in glycol methacrylate (Historesin, Leica), for light microscopy analyses, or in Paraplast (Sigma), for bromodeoxiuridine labeling and estimative of the duration of the seminiferous epithelium cycle. From the fragments included in glycol methacrylate, semi serial sections at 3 μm-thickness were obtained using a rotary microtome (Leica RM2255), observing a minimum interval of 40 μm between different cuts. The preparations were stained with toluidine blue/sodium borate 1% (Merck) and the morphometric analysis were performed using digital images captured at different magnifications with the light microscope Olympus BX-40. All images were analyzed by the software Image-Pro Plus (Media Cybernetics).

For ultrastructural analysis, testicular fragments were fixed in Karnovsky solution for 1 hour and then transferred to a 2.5% glutaraldehyde solution (EMS, Hatfield, USA) for 24 h. After being rinsed with phosphate buffer, tissues were post-fixed in 1% osmium tetroxide (EMS, Hatfield, USA) in the same buffer for 2 h. Dehydration was performed in ethanol and acetone, followed by adding embedding resin (Epon 812, EMS, Hatfield, USA). Ultrathin sections were contrasted with 3% uranyl acetate and 3% lead citrate (EMS, Hatfield, USA) and observed under a transmission electron microscope (JEOL 1011), in the Center for Microscopy and Microanalysis of UFV.

All histological processing was carefully conducted to diminish the artifacts that may be caused by histological techniques, and thus represent tissues in conditions very close to those of animals *in vivo*.

### Testicular stereology

The gonadosomatic index (GSI) was calculated dividing the testes weight by the body weight, being the value multiplied by 100.

Once the testis can be divided into the tubular and intertubular compartments: the volumetric ratios between these two were estimated by counting 3520 points projected onto 10 images captured from histological slides. Coincident points over the seminiferous tubules (tunica propria, seminiferous epithelium and lumen) and intertubule (nucleus or cytoplasm of Leydig cells, blood vessels, lymphatic spaces and connective tissue) were recorded. In order to obtaining their percentages, the counting obtained for each element in each image was divided by the number of points scored, multiplying this value by 100. Seminiferous tubules and intertubule volumes were calculated by multiplying the testes’ weight by their respective percentages and dividing these values by 100.

The tubulesomatic index (TSI) was calculated in order to quantify the investment in the seminiferous tubules regarding to the total body mass. TSI was obtained by dividing the tubular volume by the body weight and multiplying the result by 100.

The seminiferous tubules diameter and the seminiferous epithelium height were obtained measuring 20 tubular cross-sections, which presents the most circular shape regardless the stage of the cycle. The epithelium height was measured from the tunica propria to the tubular lumen.

The seminiferous tubules length (STL) per testis and per gram of testis were estimated using the formula: STL = STV/πR^2^, where STV = seminiferous tubule volume; πR^2^ = area of the seminiferous tubules cross section; and R = nuclear diameter/2; being the value obtained converted for meters. This value was divided by the testes’ weight to calculate the length of the seminiferous tubules per gram of testis.

The average diameter of the Leydig cell nucleus was obtained by counting 30 cells per animal, choosing the ones with the most spherical nuclei and evident nucleoli. The nuclear volume was obtained by the formula 4/3πR^3^, where R = nuclear diameter/2. The cytoplasmic volume was estimated by multiplying the percentage of cytoplasm by the nuclear volume, divided by the nuclear percentage. The single cell volume was estimated by adding the nuclear and cytoplasmatic volumes. These values were expressed in μm^3^.

The number of Leydig cells per testis was estimated from the Leydig cell individual volumes and the total volume occupied by Leydig cells in the testicular parenchyma. This value was divided by the testes weight, to estimate the number of Leydig cells per gram of testis, which allows comparisons between different species. The Leydigosomatic index, which quantifies the investment in Leydig cells related to body mass, was estimated by dividing the Leydig cell volume in the testicular parenchyma by the body weight, multiplied by 100.

### Stages and duration of the seminiferous epithelium cycle

The stages of the seminiferous epithelium cycle (SEC) were characterized using the tubular morphology method [15]. Eight stages were characterized based on the shape and location of the nucleus of spermatids and spermatocytes and the occurrence of figures of meiotic division. The relative frequency of the stages were calculated based on the identification and occurrence of each stage in 200 cross sections of seminiferous tubules in each animal.

To calculate the duration of the SEC, two specimens of *D*. *rotundus* were injected with 0.1 mL of commercial bromodeoxiuridine intratesticularly (Invitrogen BrdU Labeling Reagent number 00–0103 Camarillo, USA). Spermatogenesis duration can be determined via BrdU injection since this substance is incorporated into the nucleus of germ cells that are synthesizing DNA at the time of application. The animals were euthanized after 1 h and after 5 days of BrdU injection. After fixation in Karnovsky solution [14], the testes were dehydrated in a series of increasing concentrations of ethanol and subsequently cleared in three consecutive baths of xylene before being embedded in Paraplast (Sigma-Aldrich).

Detection of BrdU was done by staining sections of 4 μm thickness with a monoclonal antibody, according with the Invitrogen BrdU Staining Kit number 93–3943 (Camarillo, USA). Sections were deparaffinized and rehydrated, washed in PBS, and peroxidase activity was endogenously blocked with H_2_O_2_ and methanol. Then, the slides were washed in PBS for enzymatic pretreatment that was performed by incubation in trypsin solution. After washing in distilled water, slides were incubated with denaturing solution, followed by washing in PBS and incubation in blocking solution, which was not washed. Then, the material was incubated with biotinylated monoclonal mouse anti-BrdU, followed by the incubation with streptavidin-peroxidase. Immunoreactive cells were detected by incubating the slides with a mixture of 3,3-diaminobenzidine tetrachloride (DAB) and hydrogen peroxide, protected from light. The sections were counterstained with hematoxylin, dehydrated in ethanol, cleared in xylene and mounted.

SEC estimated duration was possible by observing the most advanced cell type in the epithelium, positive for BrdU labelling. The frequency of the stages that had gone through from the BrdU injection to its detection was then calculated. The frequency of the complete stages corresponds to the time spent, and the duration of a cycle of the seminiferous epithelium was calculated.

### Quantification of the spermatogenic yield

The population of each germ cell type was estimated by counting the germ cell nuclei and Sertoli cell nucleoli present in stage 1 of the SEC, by using 10 tubular cross-sections *per* animal. It was considered in stage 1 the cross sections which showed only one generation of spermatids at a round shape, after the second meiotic division of secondary spermatocytes [15]. The nuclear diameters of 30 type-A spermatogonia (A), primary spermatocytes in preleptotene/leptotene (PL-L), primary spermatocytes in pachytene (PC), round spermatids (RS) and nucleoli of Sertoli cells (SC) were measured. The final averages were corrected considering the cut thickness along with the nuclear or nucleolar diameters of germ cells and Sertoli cells, respectively. The cell countings were taken according to the methodology proposed by Abercrombie [16], modified by Amann and Almquist [17].

The intrinsic efficiency of spermatogenesis was calculated from the rates found among the corrected numbers of germ cells, in order to quantify the efficiency of spermatogenesis. The mitotic index, also known as coefficient of spermatogonial mitosis (PL-L/A), was determined to quantify the loss or degeneration occurring during the spermatogonial phase. The meiotic index (RS/PC) was also obtained in order to determine the efficiency of meiotic divisions and the overall spermatogenesis yield (RS/A), quantifying the efficiency of the spermatogenic process as a whole. We also determined the Sertoli cells index, which indicates the support capacity of this cell type by the total number of germ cells. This index was obtained from the rates found between the corrected number of germ cells and the corrected number of Sertoli cells in stage 1 of the SEC ((A + PL-L + PC + RS)/SC).

The total number of Sertoli cells per testis was obtained multiplying its corrected number by the tubular length per testis (in micrometers), and dividing this value by the cut thickness [18]. The number of Sertoli cells per gram of testis was obtained by dividing the total cell number by the mean testicular weight.

Assuming that the cell loss is not significant in spermiogenesis, the testicular sperm reserves (TSR), both per testis or per gram of testis, were calculated based on the round spermatids population in stage 1 of the SEC. Thus, the average number of round spermatids was taken in seminiferous tubules cross sections of known thickness. This number was corrected for the total length of the tubule per testis (in micrometers) or per gram of testis, using the following formula: TSR = (length of the seminiferous tubules/cut thickness) x corrected number of round spermatids per cross section [15]. The daily sperm production (DSP) was calculated by dividing the TSR by the SEC duration. Both the TSR and the DSP per gram of testis were obtained by dividing its values per testis by the testes’ weight.

### Statistical analysis

The results were submitted to descriptive statistical analysis and the averages obtained were expressed as mean ± standard deviation.

## Results

### Testicular stereology

**Fig 1:**
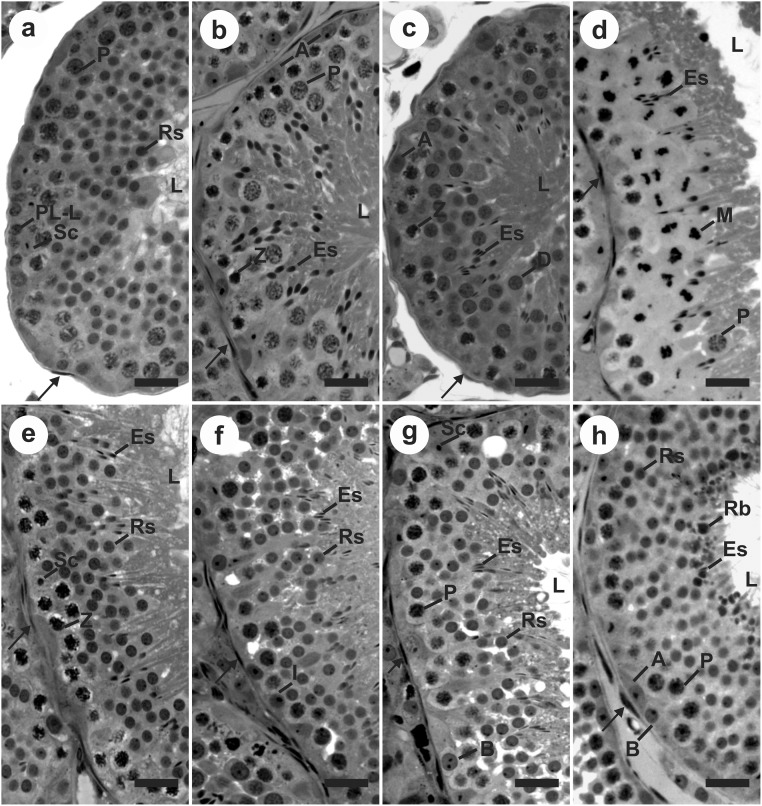
Histological cross-sections of seminiferous tubules showing the eight stages of the seminiferous epithelium cycle in *Desmodus rotundus*, according to the tubular morphology method. (a) Stage 1; (b) Stage 2; (c) Stage 3; (d) Stage 4; (e) Stage 5; (f) Stage 6; (g) Stage 7; (h) Stage 8. Sc: Sertoli cell; A: type A spermatogonia; I: intermediate spermatogonia; B: type B spermatogonia; PL-L: primary spermatocyte in preleptotene to leptotene; Z: primary spermatocyte in zygotene; P: primary spermatocyte in pachytene; D: primary spermatocyte in diplotene; M: metaphase figure; Rs: round spermatid; Es: elongating/elongated spermatid; Rb: residual bodies; L: lumen of seminiferous tubule; →: myoid cell at tunica propria. Bars: 40 μm.

**Fig 2:**
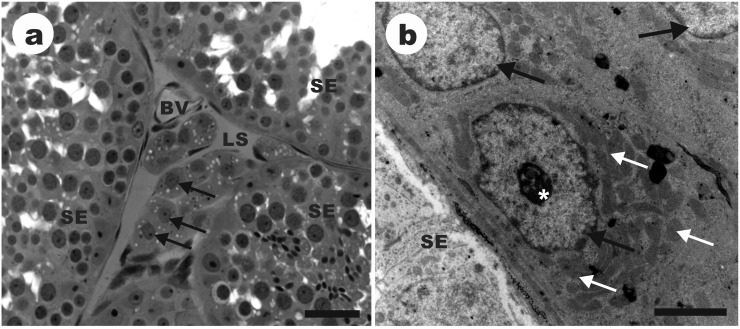
Organization of the intertubular compartment in the testis of *Desmodus rotundus*. SE: seminiferous epithelium; BV: blood vessel; LS: lymphatic space; Black arrow: Leydig cell nuclei; *: Leydig cell nucleoli; White arrowhead: lipid droplet at Leydig cell cytoplasm. White arrow: mitochondria at Leydig cell cytoplasm. Light microscopy, scale bar 30 μm (a); Transmission electron microscopy, scale bar 2 μm (b).

Biometric results and volumetric proportions of the components of the testicular parenchyma of *D*. *rotundus* are listed in Table 1, and the arrangement of testicular parenchyma are showed on Figs 1 and 2. Vampire bats showed an average body weight of 36.53g and testicular weight of 0.20g, thus resulting in a GSI of 0.54%. Discounting the percentage of 11.54% represented by albuginea, 94.09% of the testicular parenchyma was composed by seminiferous tubules and the remaining 5.91% was represented by the intertubule. With an average tubular volume of 0.18 mL and tubular diameter of 188.04 μm, the animals of this study showed tubular length of 34.70 m per gram of testis. The seminiferous epithelium, which represented 73.68% of the tubular compartment, had an average epithelium height of 56.73 μm and was obtained a TSI of 0.49%.

**Table 1:** Biometric and morphometric data of testicular components in the bat *Desmodus rotundus*.

Parameters (n = 6)	Mean±SD
**Body weight (g)**	36.53±3.32
**Testis weight (g)**	0.20±0.02
**Gonadosomatic index (%)**	0.54±0.10
**Tunica albuginea (%)**	11.54±2.77
**Testis parenchyma volume density**	
** Tubular compartment (%)**	94.09±2.16
** Tubular compartment (mL)**	0.18±0.02
** Tunica propria (%)**	3.35±0.38
** Seminiferous epithelium (%)**	73.68±5.18
** Lumen (%)**	17.05±3.60
** Intertubular compartment (%)**	5.91±2.16
** Intertubular compartment (mL)**	0.013±0.005
** Leydig cell (%)**	51.20±20.16
** Connective tissue (%)**	6.04±4.12
** Blood vessel (%)**	19.5±4.36
** Lymphatic space (%)**	23.25±14.88
**Tubular diameter (μm)**	188.04±35.96
**Seminiferous epithelium height (μm)**	56.73±11.72
**Tubular length per testis (m)**	6.96±3.28
**Tubular length per gram of testis (m)**	34.70±14.16
**Tubulesomatic index (%)**	0.49±0.10

Data are expressed as mean ± standard deviation.

While the seminiferous epithelium was the main component of the tubular compartment (Fig 1), the intertubular compartment was composed predominantly by Leydig cells (Fig 2), followed by lymphatic space (Table 1). Lipid droplets could be observed on the cytoplasm of Leydig cells (Fig 2a), and the mitochondria were the main organelle (Fig 2b). These cells showed an average nuclear diameter of 7.18 μm, leading to a nuclear, cytoplasmic and total volume of Leydig cell of 196.96 μm^3^, 856.98 μm^3^ and 1053.88 μm^3^, respectively. The average number of Leydig cells *per* gram of testis was 23.77x10^6^ cells, and the Leydigosomatic index was 0.015% (Table 2).

**Table 2:** Leydig cell morphometry in the bat *Desmodus rotundus*.

Parameters (n = 6)	Mean±SD
**Nuclear diameter (μm)**	7.18±0.57
**Nuclear percentage (%)**	20.31±7.34
**Cytoplasm percentage (%)**	79.69±7.34
**Nucleus volume (μm**^**3**^**)**	196.96±50.66
**Cytoplasm volume (μm**^**3**^**)**	856.98±384.98
**Leydig cell volume (μm**^**3**^**)**	1053.88±388.55
**Leydig cell number *per* testis (x10**^**5**^**)**	46.90±19.30
**Leydig cell number *per* gram of testis (x10**^**6**^**)**	23.77±8.77
**Leydigosomatic index (%)**	0.015±0.009

Data are expressed as mean ± standard deviation.

### Stages of the seminiferous epithelium cycle

**Fig 3:**
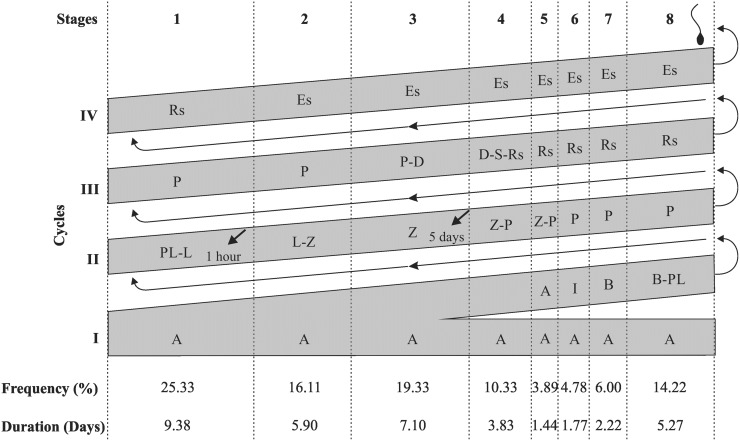
Diagram of the spermatogenic process in *Desmodus rotundus*, with the mean frequency (%) and the duration in days of each one of the eight stages of the seminiferous epithelium cycle. Each line corresponds to a generation of spermatogenic cells and each column corresponds to one stage. The roman numerals indicate the spermatogenic cycles. A: type A spermatogonia; I: intermediate spermatogonia; B: type B spermatogonia; PL-L: primary spermatocyte in preleptotene to leptotene; Z: primary spermatocyte in zygotene; P: primary spermatocyte in pachytene; D: primary spermatocyte in diplotene; S: secondary spermatocyte; Rs: round spermatid; Es: elongated spermatid. The labeled germinative cells which was more advanced (arrows) at the eight stages of the cycle, 1 hour and 5 days after treatment with bromodeoxiuridine, was respectively the PL-L at stage 1 and the Z at stage 3.

Fig 1 shows the 8 stages comprising the seminiferous epithelium cycle (SEC) of *D*. *rotundus*, according to the tubular morphology method, and the progression of cell divisions and cell specific associations in each stage are schematized in Fig 3.

Sertoli cells and type A spermatogonia were observed in all stages. During the spermatogonial mitosis the A-type spermatogonia undergoes transition to the Intermediate-type, at stage 6, while B-type spermatogonia are at stage 7. B-type spermatogonia originates the primary spermatocyte in pre-leptotene at stage 8, and this cell type starts the first meiotic division. It will be in the transition from pre-leptotene to leptotene on the next stage 1. The primary spermatocyte in leptotene will be found in the stages 1 to 2, originating the spermatocyte in zygotene at stage 2. This was also found in stage 3 and undergoes transition to the pachytene on stages 4 and 5. Primary spermatocytes in pachytene were found at stages 5 to 8. This cell type undergoes transition to the diplotene at the following stage 3, and the second meiotic division happens on the following stage 4, originating secondary spermatocytes. Since the second meiotic division is faster than the first, the secondary spermatocyte quickly originates the round spermatids, still at stage 4. These newly formed round spermatids initiate the elongating process in the next stage 2, and elongated spermatids can be seen from stages 3 to 8.

Therefore, different generations of spermatocytes and only one generation of spermatids are usually observed in stages 1 to 3, while one generation of spermatocytes and two generations of spermatids could be observed from stages 5 to 8. These patterns are due to the two meiotic divisions occurring at stage 4, when we observed spermatocytes in the metaphase plate (Fig 1d). This stage is characterized by the transition from primary spermatocytes in diplotene to secondary spermatocytes, and from these to round spermatids. From stages 5 to 8, both newly formed round spermatids (old generation) and the elongated spermatids can be seen. Elongated spermatids became more and more elongated and close to the tubular lumen as we observed them at a developmental stage ready to be released from the seminiferous epithelium (Figs 1 and 3).

According to the average frequencies represented by each stage (Fig 3), the pre-meiotic, meiotic and post-meiotic stages represented respectively 60.78%, 10.33% and 28.89% of the SEC.

### Duration of the seminiferous epithelium cycle

**Fig 4:**
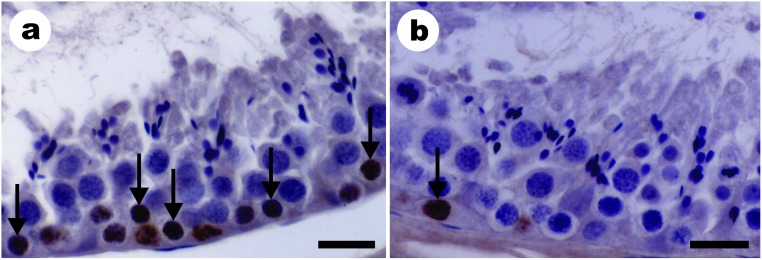
The most advanced labelled germ cell types found after intratesticular bromodeoxiuridine injections in *Desmodus rotundus*. Leptotene to Zygotene cells in stage 3 (a) and type A spermatogonia in stage 3 to 4 (b) of the seminiferous epithelium cycle. Arrows indicate marking with bromodeoxiuridine. Bars: 20 μm.

Stained cells, most advanced in the seminiferous epithelium after 1 hour of BrdU application, were the primary spermatocytes in preleptotene-leptotene at stage 1 of the seminiferous epithelium cycle (SEC) (Fig 3). The most advanced labelled cells after 5 days of BrdU application was the primary spermatocyte, ending the transition from leptotene to zygotene at stage 3 (Figs 3 and 4a). During the transition from stage 3 to 4, only the spermatogonia remained labeled (Fig 4b). Within 5 days, we observed a 60.78% progression of the SEC (average frequency of the stages 1 to 3). A cycle was therefore 8.23 days on average. Considering that 4.5 seminiferous epithelium cycles are necessary for the whole spermatogenic processes to be completed, we estimate a total of 37.02 days for the complete spermatogenesis duration in *D*. *rotundus*.

### Quantification of the spermatogenic yield

The counting of germ cells at stage 1 of SEC are shown in Table 3. These counting showed a mitotic index of 16.93%, meiotic index of 2.51% and spermatogenic yield of 60.00%. Considering all germ cells, a single Sertoli cell of *D*. *rotundus* is able to support 20.06 germ cells. Each testis of *D*. *rotundus* showed an average of 13.20x10^6^ Sertoli cells and in each gram of testis were found on average 13.10x10^7^ Sertoli cells. The spermatic reserve found in each gram of testis was of 71.44x10^7^ cells, and the daily spermatic production per gram of testis was of 86.80x10^6^ spermatozoon.

**Table 3:** Germ cells and spermatic production indexes in *Desmodus rotundus*.

Parameters (n = 6)	Mean±SD
Cell population at stage 1 of the SEC*	
** Type A spermatogonia**	1.21±0.44
** Pre-leptotene/leptotene spermatocyte**	18.92±4.55
** Pachytene spermatocyte**	26.47±4.74
** Round spermatid**	65.90±8.29
** Sertoli cell**	5.76±0.80
**Mitotic index**	16.93±6.45
**Meiotic index**	2.51±0.18
**Spermatogenic yield**	60.00±22.12
**Sertoli cell index**	20.06±5.44
**Sertoli cell number per testis (x 10**^**6**^**)**	13.20±6.20
**Sertoli cell number per gram of testis (x 10**^**7**^**)**	13.10±5.42
**Spermatic reserve per testis (x 10**^**7**^**)**	14.20±4.52
**Spermatic reserve per gram of testis (x 10**^**7**^**)**	71.44±17.94
**Daily spermatic production per testis (x 10**^**6**^**)**	17.25±5.49
**Daily spermatic production per gram of testis (x 10**^**6**^**)**	86.80±21.80

* SEC = seminiferous epithelium cycle, according to the tubular morphology method [15].

### Ultrastructure of germ cells

**Fig 5:**
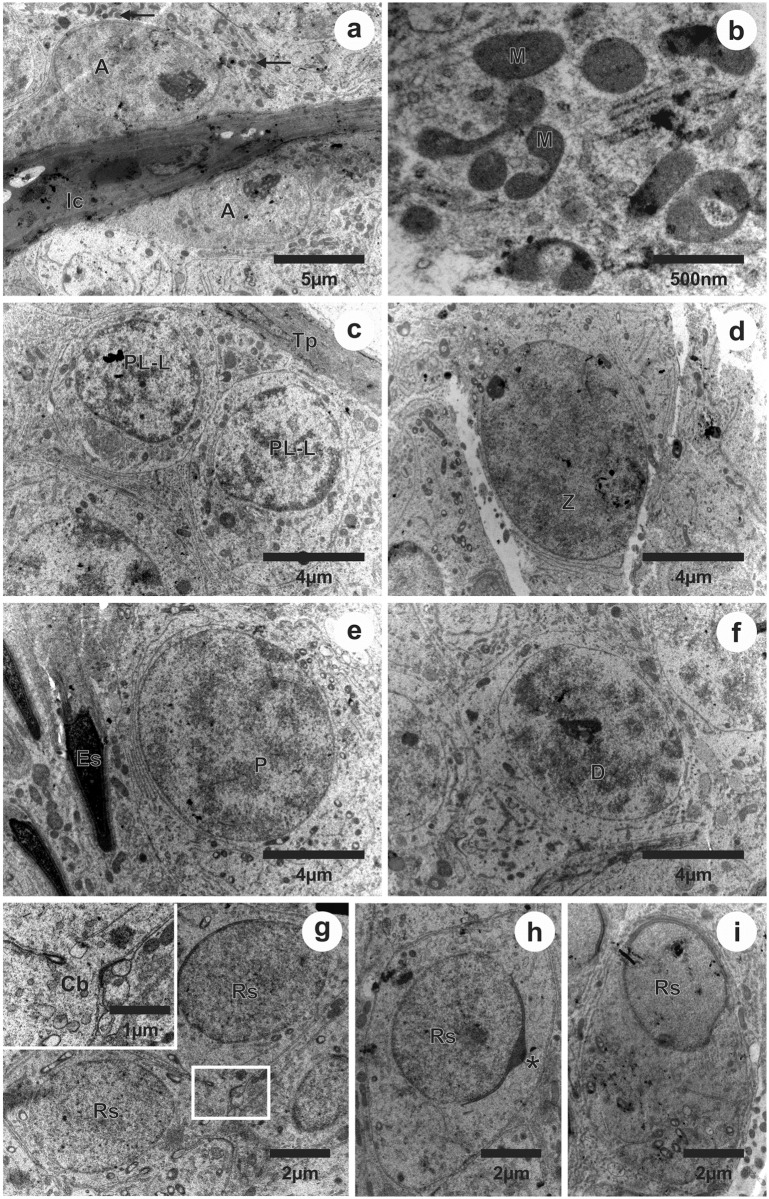
Ultrastructure of the germ cells of the seminiferous epithelium in *Desmodus rotundus*. A: type A spermatogonia; PL-L: primary spermatocyte in preleptotene to leptotene; Z: primary spermatocyte in zygotene; P: primary spermatocyte in pachytene; D: primary spermatocyte in diplotene; Rs: round spermatid; Es: elongated spermatid; Tp: tunica propria; Ic: intertubular compartment; M and →: mitochondria; Cb: cytoplasmic bridge; *: Acrosomal cap.

The ultrastructure of some cell types of the seminiferous epithelium is shown in Fig 5. We observed the spermatogonia located near the basal lamina of the epithelium (Fig 5a). In the same region, we observed a large amount of scattered mitochondria in Sertoli cells’ cytoplasm (Fig 5b). Primary spermatocytes at the transition from pre-leptotene to leptotene were also observed near the basal lamina (Fig 5c). The leptotene spermatocytes originate zygotene spermatocytes (Fig 5d), which originate pachytene spermatocytes (Fig 5e) and finally diplotene spermatocytes (Fig 5f). Gradually these cell types advanced in the seminiferous epithelium into the tubular lumen. Surrounding the lumen we found the round spermatids (Fig 5g–5i) with cytoplasmic bridges (Fig 5g) and elongated spermatids, which were initially inserted into the seminiferous epithelium and ascended gradually from stages 5 to 8 (Fig 5e). The two generations of spermatids could be distinguished by its nuclear shape and acrosomal formation (Fig 5e and 5h).

## Discussion

Considering the worldwide distribution and great diversity exhibited by bats, remarkably limited attention has been given to different patterns of reproduction in males. Overall, the reproductive system characteristics are not well described, and little is known about the control of reproduction [1], specially at a cellular level. Given the lack of information for this particular Order, we provide here a better understanding of the spermatogenic process in one of the most interesting and intriguing bat species—the common vampire bat.

### Testicular stereology

When compared to other bat species, *D*. *rotundus* GSI was higher than the index observed in the frugivorous bat *Sturnira lilium* (0.27%, [12]) and in the insectivorous bat *Molossus molossus* (0.46%, [10]). It is proposed that the testicular size is closely related to the species reproductive behavior [19]. Most Neotropical bats live in single male-multifemale groups (harems) and usually have higher GSI compared to monogamous species, such as the crab-eating fox (0.06%, [20]). Higher GSI found in these species reflects a higher testicular investment required for the maintenance of the harem [1].

The percentages of testicular parenchyma in seminiferous tubules and intertubule were similar to the observed in the bats *Artibeus lituratus* [21], *S*. *lilium* [12] and *M*. *molossus* [10], while the percentages of tunica propria, seminiferous epithelium and lumen found for vampire bats were similar to that observed in *M*. *molossus* [10]. The tubular diameter and the height of seminiferous epithelium were larger than observed in other bats [10, 12].

As for the GSI, the higher TSI and length of seminiferous tubules *per* gram of testis found in *D*. *rotundus*, as well in other bats [10, 12, 21], seems to be associated to their reproductive behavior. It is proposed that smaller animals show a higher investment in sperm production [19]. The higher tubular length is also in accordance with the pattern previously described for other bats [10, 12, 21], which is in agreement with the hypothesis that bats usually show a tubular length considerably larger than most mammals [22–26].

Leydig cells were the main component in the intertubular space, which also follows the pattern previously described for other bats. In vampire bat, the percentage of Leydig cells was lower compared to frugivorous and insectivorous bats and consequently the percentage of lymphatic space was larger [21, 27, 28], yet within the numbers reported for other mammals [22, 29–33]. The larger investment in Leydig cells found in bats might also be associated to their reproductive behavior, as high levels of testosterone would be required for the maintenance of spermatogenesis and male dominance in harem colonies [23, 27, 28]. This steroidogenic capacity is reinforced by the large number of mitochondria in their cytoplasm, being that the shape of this organelle frequently varies from spherical to elongated or tubular in steroidogenic cells [34].

The results obtained from the Leydig cells morphometry were also bellow the observed in *M*. *molossus* [27] and in *S*. *lilium* [28], however higher than in *Myotis levis* [35] and within the recorded for other mammals [22, 29–33]. The Leydigosomatic index was similar to that observed in other bats and higher than the observed in larger animals [21, 27, 28, 30, 32, 33].

### Stages and duration of the seminiferous epithelium cycle

In *Desmodus*, the 8 stages comprising the seminiferous epithelium cycle (SEC), according to the tubular morphology method [15], followed the pattern found in other bats and mammals, as described further. Stage 1 was the most frequent, which is in accordance with the observed in several mammals [10, 11, 22, 23, 30, 36–39]. Unlike *M*. *molossus* and *S*. *lilium*, *D*. *rotundus* testes showed primary spermatocytes in zygotene at stage 2, similarly the pattern described in other mammalian orders [10, 11, 22, 26, 40–41].

In general, the duration of the spermatogenic cycle is constant among individuals from the same species, since it is under the germ cells genotype control [42]. The average duration of 8.23 days for each cycle of SEC found in *D*. *rotundus* was within the range of 7 to 14 days registered for other mammals [22]. The shortest duration was registered for the bat *S*. *lilium*, with 3.45 days, and the largest duration was registered for the opossum *Didelphis albiventris*, with 17.30 days [11, 43]. Total duration of spermatogenesis in *D*. *rotundus* (37.02 days) was also within the average previously recorded for mammals, varying from 29 to 75 days [22, 24, 44, 45]. We emphasize, however, that the values we obtained for vampire bats were closer to the lower values recorded. For this reason, its short spermatogenic cycle seems to allow males to regulate their reproductive cycle in accordance with the sexual readiness of females, described as polyestrous. These data corroborate so with previous findings in relation to the male reproductive behavior [1].

### Spermatogenic yield

The mitotic index shows that 16.93 primary spermatocytes in pre-leptotene to leptotene were produced by each type A spermatogonia, thus estimating a cell loss of 74% at the spermatogonial phase. This percentage loss is within the range of 60 to 90% previously registered for mammals [10, 12, 22]. The meiotic yield shows that 2.5 round spermatids were produced at the end of meiosis, also within the range recorded for mammals (from 1.90 to 3.62 cells) [10, 12, 22, 26, 40, 46–51]. The overall yield of spermatogenesis estimated for *D*. *rotundus* was 60 spermatozoa, below the number recorded for *S*. *lilium* and above the annual average recorded for *M*. *molossus* and several other domestic and wild mammals [10, 12, 22, 26, 40, 49, 50].

The support capacity performed by Sertoli cells, which was 20.06 germ cells in *D*. *rotundus*, could be considered high, and was within the average of other mammals, ranging from 10 to 22 cells [22, 26, 49, 52, 53], in contrast to the low capacity support observed in *S*. *lilium* [12] and *M*. *molossus* [10]. Similar to *M*. *molossus*, we observed a large number of Sertoli cells per gram of testis in *D*. *rotundus* when compared to other mammals [10, 22, 26, 51]. This cell interacts directly with the germ cells and provides several functions that are crucial for the regulation of spermatogenesis, like the support and nutrition of the developing germ cells, immunological protection for the haploid cells and release of late spermatids into the tubular lumen [22, 54].

The spermatic reserve found per gram of testis in *D*. *rotundus* was similar to that observed in other bats, which was found to be between 56.64x10^7^ and 76.52x10^7^ cells [10, 12]. This reserve is higher in bats as compared to larger animals, as the jaguar and the African lion, found to have among 103.80x10^6^ and 165.90x10^6^ cells [47, 50]. Regarding the daily spermatic production per gram of testis in *D*. *rotundus*, that value was 2.4 times smaller to that found in *S*. *lilium* and three times higher than in mammals such as the jaguar, paca and agouti [46, 55, 56], corroborating the hypothesis of higher investment in spermatic production in smaller animals [19].

### Ultrastructure of germ cells

The ultrastructure of germ cells followed the pattern found in other mammals, including other bat species. Were observed type A spermatogonia and primary spermatocytes in transition from pre-leptotene to leptotene in its characteristic location, closer to the epithelium basement membrane. During the meiotic phase, the primary spermatocyte begins to move away from the basement membrane into the tubular lumen, leaving the baseline setting and occupying the adluminal compartment [12, 54, 57, 58].

From the spermatocyte in leptotene emerges the synaptonemal complex, which is present until the diplotene phase. Gradually the nucleus increases in size and the chromatin becomes condensed. Nucleolar structure also becomes condensed and well organized in leptotene and zygotene phases, losing its morphological integrity during pachytene and diplotene. In general, spermatocytes are cells of sparse cytoplasm and a few organelles, and mitochondrias are the most frequently observed [54, 57, 58].

Despite the spermatids can be seen in all stages, they can be distinguished by their nuclear shape and acrosome formation, which occupies about 95° to 120° of the nuclear surface in the first generation of spermatid, the round spermatids, at stage 7 of the SEC, and are in complete formation over the nuclear surface of the second generation of spermatids, the elongated spermatid, from the stages 6 to 8 of the SEC [59]. The cytoplasmic bridges evidenced between the round spermatids allow a synchronous cellular development, as the various cell generations observed in the seminiferous epithelium are in a sequence of increasing maturation, initiating from the base to the tubular lumen [54].

## Conclusions

Considering our findings, we conclude that *D*. *rotundus* shows a testicular pattern similar to that described for other mammals. The results obtained on testicular morphometry and indicative indices of sperm production were generally similar to those described in other bats species. In this study we corroborate the trend proposal of high sperm production found through spermatogenic studies of other chiropterans. Our results are also in agreement with previous findings describing a continuous reproductive cycle in tropical bats, directly related to females polyestry and the promiscuous mating system. Additionally, the first description of the total spermatogenesis duration in *D*. *rotundus* may be considered an important tool for studies aiming at an adequate reproductive management of the species in order to minimize damage to public health and livestock, regarding the transmission of the rabies virus, without affecting the conservation status of this species.

## References

[1] Crichton EG, Krutzsch PH. Reproductive biology of bats In: Racey PA, Entwistle AC (eds.), Life-history and Reproductive Strategies of Bats, London: Academic Press; 2000:364–367.

[2] Greenhall AM, Joermann G, Schmidt U, Seidel MR. Desmodus rotundus Mammalian Species, New York: The American Society of Mammalogists; 1983:1–6.

[3] Acha PN, Malaga Alba A. Economic losses due to *Desmodus rotundus* In: Greenhall AM, Schmidt U (eds.), Natural history of vampires bats, Florida: CRC Press Inc; 1988:207–214.

[4] Mayen F. Haematophagus bats in Brazil, their role on rabies transmission, impact on public health, livestock industry and alternatives to an indiscriminate beduction of bat population. J Vet Med B Infect Dis Vet Public Health 2003; 50:469–472. 14720182 10.1046/j.1439-0450.2003.00713.x

[5] Wilkinson GS. The social organization of the common vampire bat, I. Pattern and cause of association. Behav Ecol Sociobiol 1985; 17:111–121.

[6] Taddei VA, Gonçalves CA, Pedro WA, Tadei WJ, Kotait I, Arieta C. Distribuição do morcego vampiro *Desmodus rotundus* no Estado de São Paulo e a raiva dos animais domésticos, Campinas: Impresso Especial da CATI; 1991:107.

[7] Alencar AO, Silva GAP, Arruda MM, Soares AJ, Guerra DQ. Aspectos biológicos e ecológicos de *Desmodus rotundus* (Chiroptera) no nordeste do Brasil. Pesq Vet Bras 1994; 14(4):95–103.

[8] Orsi AM, Vicentini CA, Dias SM, Michelin SC, Viotto MJ. Histochemical and structural characteristics of the testis of the vampire bat (*Desmodus rotundus rotundus*, Geoffrey, 1810). Rev Bras Biol 1990; 50(1):221–228. 2089486

[9] Orsi AM, Vicentini CA, Dias SM, Vicentini IBF, Moreno MH. Características ultraestruturais das células de Sertoli do morcego hematófago (*Desmodus rotundus rotundus*, Geoffrey, 1810). Rev Bras Biol 1993; 53:583–590. 8209033

[10] Morais DB, Cupertino MC, Goulart LS, Freitas KM, Freitas MB, Paula TA, et al Histomorphometric evaluation of the *Molossus molossus* (Chiroptera, Molossidae) testis: The tubular compartment and indices of sperm production. Anim Reprod Sci 2013; 140:268–278. 10.1016/j.anireprosci.2013.06.003 23845822

[11] Morais DB, Paula TAR, Barros MS, Balarini MK, Freitas MB, Matta SLP. Stages and duration of the seminiferous epithelium cycle in the bat *Sturnira lilium* (E. Geoffroy, 1810, Chiroptera: Phyllostomidae). J Anat 2013; 3:372–379. 10.1111/joa.12016 PMC3582256 23305159

[12] Morais DB, Barros MS, Paula TA, Freitas MB, Gomes ML, Matta SL. Evaluation of the cell population of the seminiferous epithelium and spermatic indexes of the bat *Sturnira lilium* (Chiroptera: Phyllostomidae). PLoS One 2014; 9(7):e101759 10.1371/journal.pone.0101759 25003782 PMC4086963

[13] Kunz TH, Anthony ELP. Age estimation and post-natal growth in the bat *Myotis lucifugus*. J Mammal 1982; 63:23–32.

[14] Karnovsky MJ. A formaldehyde-glutaraldehyde fixative of high osmolarity for use in electron microscopy. J Cell Biol 1965; 27:137A.

[15] Berndtson WE. Methods for quantifying mammalian spermatogenesis: a review. J Anim Sci 1977; 44(5):818–883. 324963 10.2527/jas1977.445818x

[16] Abercrombie M. Estimation of nuclear population from microtome sections. Anat Rec 1946; 94:239–247. 21015608 10.1002/ar.1090940210

[17] Amann RP, Almquist JO. Reproductive capacity of dairy bulls.VIII. Direct and indirect measurement of testicular sperm production. J Dairy Sci 1962; 45:774–781.

[18] Neves ES, Chiarini-Garcia H, França LR. Comparative testis morphometry and seminiferous epithelium cycle length in donkeys and mules. Biol Reprod 2002; 247–255. 12080024 10.1095/biolreprod67.1.247

[19] Kenagy GJ, Trombulak SC. Size and function of mammalian testes in relation to body size. J Mammal 1986; 67(1):1–22.

[20] Caldeira BC, Paula TAR, Matta SLP, Balarini MK, Campos PKA. Morphometry of testis and seminiferous tubules of the adult crab-eating fox (*Cerdocyon thous*, Linnaeus, 1766) adulto. Ceres 2010; 57(5):569–575.

[21] Miranda DC. Efeito dos fungicidas Mancozeb e Tebuconazol sobre parâmetros testiculares do morcego frugívoro Artibeus lituratus (Olfers, 1818). Viçosa, BR: Universidade Federal de Viçosa; 2012. Dissertation.

[22] França LR, Russell LD. The testis of domestic mammals In: Martinez-Garcia F, Regadera J (eds.), Male Reproduction: A Multidisciplinary Overview, Madrid: Churchill Livingstone; 1998:197–219.

[23] Paula TAR, Costa DS, Matta SLP. Avaliação histológica quantitativa do testículo de capivaras (*Hydrochoerus hydrochaeris*) adultas. J Biosci 2002; 18(1):121–136.

[24] Leal MC, França LR. The seminiferous epithelium cycle length in the black tufted-ear marmoset (*Callithrix penicillata*) is similar to humans. Biol Reprod 2006; 74:616–624. 10.1095/biolreprod.105.048074 16319285

[25] Bittencourt VL, Paula TAR, Matta SLP, Fonseca CC, Costa DS, Costa EP, et al Biometria macro e microscópica dos componentes testiculares em lobo guará (*Chrysocyon brachyurus*, Illiger,1811) adulto. Ceres 2007; 54(313):329–340.

[26] Costa KLC, Matta SLP, Gomes MLM, Paula TAR, Freitas KM, Carvalho FAR, et al Histomorphometric evaluation of the neotropical brown brocket deer *Mazama gouazoubira* testis, with an emphasis on cell population indexes of spermatogenic yield. Anim Reprod Sci 2011; 127(3–4):202–212. 10.1016/j.anireprosci.2011.07.016 21889273

[27] Morais DB, Oliveira LC, Cupertino MC, Freitas KM, Freitas MB, Paula TA, et al Organization and seasonal quantification of the intertubular compartment in the bat *Molossus molossus* (Pallas, 1776) testis. Microsc Res Tech 2013; 76(1):94–101. 10.1002/jemt.22141 23077089

[28] Morais DB, Barros MS, Freitas MB, Paula TA, Matta SL. Histomorphometric characterization of the intertubular compartment in the testes of the bat *Sturnira lilium*. Anim Reprod Sci 2014; 147(3–4):180–186. 10.1016/j.anireprosci.2014.03.008 24793584

[29] França LR, Godinho CL. Testis morphometry, seminiferous epithelium cycle length, and daily sperm production in domestic cats (*Felis catus*). Biol Reprod 2003; 68(5):1554–1561. 10.1095/biolreprod.102.010652 12606460

[30] Barros JBG. Análise morfofuncional do testículo e da espermatogênese de leões africanos (Panthera leo, Linnaeus, 1758) adultos. Viçosa, BR: Universidade Federal de Viçosa; 2005. Thesis.

[31] Costa DS, Paula TAR, Matta SLP. The intertubular compartment morphometry in capybaras (*Hydrochoerus hydrochaeris*) testis. Anim Reprod Sci 2006; 91:173–179. 10.1016/j.anireprosci.2005.03.013 16310104

[32] Azevedo MHF, Paula TAR, Balarini MK, Matta SLP, Peixoto JV, Guião-Leite FL, et al Organization and quantification of the elements in the intertubular space in the adult jaguar testis (*Panthera onca*). Micron 2008; 39 (8):1166–1170. 10.1016/j.micron.2008.05.005 18602267

[33] Sarti P, Paula TAR, Polli GO, Deco-Souza T, Araujo GR. Morfofisiologia do tecido intertubular e das células de Leydig de jaguatirica (*Leopardus pardalis*) adulta. Arq Bras Med Vet Zootec 2011; 63(5):1060–1065.

[34] Duarte A, Poderoso C, Cooke M, Soria G, Cornejo Maciel F, Gottifredi V, et al Mitochondrial fusion is essential for steroid biosynthesis. PLoS One 2012; 7(9):e45829 10.1371/journal.pone.0045829 23029265 PMC3448708

[35] Farias TO, Notini AA, Talamoni SA, Godinho HP. Testis morphometry and stages of the seminiferous epithelium cycle in an epididymal sperm-storing neotropical vespertilionid, *Myotis levis* (Chiroptera). Anat Histol Embryol 2014; 44(5):361–369. 10.1111/ahe.12148 25258091

[36] Guião-Leite FL, Paula TAR, Matta SLP, Fonseca CC, Neves MTD, Barros JBG. Cycle and duration of the seminiferous epithelium in puma (*Puma concolor*). Anim Reprod Sci 2006; 91:307–316. 10.1016/j.anireprosci.2005.04.003 15923093

[37] Bittencourt VL, Paula TAR, Matta SLP, Fonseca CC, Benjamin LA, Costa DS. The seminiferous epithelium cycle and daily spermatic production in the adult maned wolf (*Chrysocyon brachyurus*, Illiger, 1811). Micron 2007; 38(6):584–589. 10.1016/j.micron.2006.10.004 17157026

[38] Beguelini MR, Moreira PRL, Faria KC, Marchesin SRC, Morielle-Versute E. Morphological characterization of the testicular cells and seminiferous epithelium cycle in six species of neotropical bats. J Morphol 2009; 270(8):943–953. 10.1002/jmor.10731 19248152

[39] Balarini MK, Paula TAR, Matta SLP, Peixoto JV, Guião-Leite FL, Rossi-Junior JL, et al Stages and duration of the cycle of the seminiferous epithelium in oncilla (*Leopardus tigrinus*, Schreber, 1775). Theriogenol 2011; 77(5):873–880. 10.1016/j.theriogenology.2011.09.011 22153265

[40] Morais ACT, Balarini MK, Lopes EO, Menezes TP, Quintela FM, Morais DB, et al The tubular compartment and the spermatogenic dynamics of the wild rodent *Oxymycterus nasutus* (Rodentia: Cricetidae). Anim Reprod Sci 2014; 149(3–4):249–258. 10.1016/j.anireprosci.2014.06.027 25037444

[41] França LR, Ogawa T, Avarbock MR, Brinster RL, Russell LD. Germ cell genotype controls cell cycle during spermatogenesis in the rat. Biol Reprod 1998; 59:1371–1377. 9828180 10.1095/biolreprod59.6.1371

[42] Paula TAR, França LR, Chiarini-Garcia H. Seminiferous epithelium cycle and its duration in capybaras (*Hydrochoerus hydrochaeris*). Tissue Cell 1999; 31(3): 327–334. 10.1054/tice.1999.0039 10481304

[43] Queiroz GF, Nogueira JC. Duration of the cycle of the seminiferous epithelium and quantitative histology of the testis of the South American white-belly opossum (*Didelphis albiventris*), Marsupialia. Reprod Fertil Dev 1992; 4:213–222. 1438950 10.1071/rd9920213

[44] Hess RA, França LR. Spermatogenesis and cycle of the seminiferous epithelium In: Cheng CY (ed.), Molecular Mechanisms in Spermatogenesis. New York: Landes Bioscience; 2007:1–15.

[45] Morais ACT, Balarini MK, Menezes TP, Ferraz FS, Gomes MLM, Morais DB, et al 2016 Germ cells and the seminiferous epithelium cycle in the wild rodent *Oxymycterus rufus* (Rodentia: Cricetidae). J Pharm Biol Sci 2016; 11(4): 61–71.

[46] Guião-Leite FL, Paula TAR. Rendimento intrínseco da espermatogênese, o índice de células de Sertoli e a produção espermática diária da onça parda (*Puma concolor*). Rev Bras Reprod Anim 2003; 27(1):21–26.

[47] Bittencourt VL, Paula TAR, Matta SLP, Fonseca CC, Neves MTD, Costa MEL, et al Avaliação da população celular do epitélio seminífero e índices indicativos da produção espermática, através de biópsia testicular em lobo-guará (*Chrysocyon brachyurus*, IIiger 1811) adulto. Rev Bras Rep Anim 2004; 28(2):108–113.

[48] Barros JBG, Paula TAR, Matta SLP, Fonseca CC, Guião-Leite FL, Rossi-Jr JL, et al Sertoli cell index and spermatic reserves in adult captive African lions (*Panthera leo*, Linnaeus,1758). Anim Reprod Sci 2007; 102(3–4):350–356. 10.1016/j.anireprosci.2007.04.002 17499460

[49] Costa DS, Menezes CMC, Paula TAR. Spermatogenesis in white-lipped peccaries (*Tayassu pecari*). Anim Reprod Sci 2007; 98(3–4):322–334. 10.1016/j.anireprosci.2006.03.014 16647229

[50] Azevedo MHF, Paula TAR, Matta SLP, Fonseca CC, Costa EP, Costa DS, et al Cell population indexes of spermatogenic yield and testicular sperm reserves in adult jaguars (*Panthera onca*). Anim Reprod Sci 2010; 118(1):83–88. 10.1016/j.anireprosci.2009.05.018 19564086

[51] Melo FCSA, Matta SLP, Paula TAR, Gomes MLM, Oliveira LC. The effects of *Tynnanthus fasciculatus* (Bignoniaceae) infusion on testicular parenchyma of adult Wistar rats. Biol Res 2010; 43:445–450. doi: /S0716-97602010000400009 21526271

[52] Zhengwei Y, Wreford NG, Royce P, Kretser D, McLachlan RI. Stereological evaluation of human spermatogenesis after suppression by testosterone treatment: heterogeneous pattern of spermatogenic impairment. J Clin Endocrinol Metab 1998; 83(4):1284–1291. 10.1210/jcem.83.4.4724 9543157

[53] Zhengwei Y, McLachlan RI, Bremmer WJ, Wreford NG. Quantitative (stereological) study of the normal spermatogenesis in the adult monkey (*Macaca fascicularis*). J Androl 1997; 18:681–687. 9432141

[54] Russell LD, Ettlin RA, Sinha-Hikim AP, Clegg ED. Histological and histopathological evaluation of the testis. Clearwater: Cache River Press; 1990.

[55] Costa GMJ, Chiarini-Garcia H, Morato RG, Alvarenga RLLS, França LR. Duration of spermatogenesis and daily sperm production in the jaguar (*Panthera onca*). Theriogenol 2008; 70:1136–1146. 10.1016/j.theriogenology.2008.06.035 18672284

[56] Costa GMJ, Leal MC, Silva JV, Ferreira ACS, Guimarães DA, França LR Spermatogenic cycle length and sperm production in a feral pig species (Collared Peccary, *Tayassu tajacu*). J Androl 2010; 31:221–230. 10.2164/jandrol.109.008524 19745218

[57] Beguelini MR, Puga CCI, Taboga SR, Morielle-Versute E. Ultrastructure of spermatogenesis in the white-lined broad-nosed bat, *Platyrrhinus lineatus* (Chiroptera: Phyllostomidae). Micron 2011; 42:586–599. 10.1016/j.micron.2011.02.004 21458280

[58] Beguelini MR, Taboga SR, Morielle-Versute E. Ultrastructural characteristics of spermatogenesis in Pallas’s Mastiff Bat, *Molossus molossus* (Chiroptera: Molossidae). Micros Res Tech 2012; 75:856–868. 10.1002/jemt.22005 22253210

[59] Morais DB, Paula TAR, Freitas KM, Matta SLP. Cycle of the seminiferous epithelium of the bat *Molossus molossus*, characterized by tubular morphology and acrosomal development. Asian Pac J Reprod 2012; 1(4): 303–307.

